# Incarceration experiences among a community-recruited sample of injection drug users in Bangkok, Thailand

**DOI:** 10.1186/1471-2458-9-492

**Published:** 2009-12-30

**Authors:** Kanna Hayashi, M-J Milloy, Nadia Fairbairn, Karyn Kaplan, Paisan Suwannawong, Calvin Lai, Evan Wood, Thomas Kerr

**Affiliations:** 1 Urban Health Research Initiative, British Columbia Centre for Excellence in HIV/AIDS, Vancouver, Canada; 2 School of Population and Public Health, University of British Columbia, Vancouver, Canada; 3 Mitsampan Harm Reduction Center/Thai AIDS Treatment Action Group, Bangkok, Thailand; 4 Department of Medicine, University of British Columbia, Vancouver, Canada

## Abstract

**Background:**

Since 2003 Thailand has waged an aggressive "war on drugs" campaign focused on arresting and incarcerating suspected drug users and dealers. However, little is known about incarceration experiences among IDU in the wake of the recent war on drugs. Therefore, we sought to examine incarceration experiences among IDU in Bangkok, Thailand.

**Methods:**

We examined the prevalence of incarceration among community-recruited IDU participating in the Mitsampan Community Research Project. Multivariate logistic regression was used to identify factors associated with a self-reported history of incarceration. We also examined the prevalence of injection drug use and syringe sharing within prisons.

**Results:**

252 IDU were recruited in August 2008; 66 (26.2%) were female and the median age was 36.5 years. In total, 197 (78.2%) participants reported a history of incarceration. In multivariate analyses, reporting a history of incarceration was associated with a history of compulsory drug treatment (adjusted odds ratio [AOR] = 4.93; 95% confidence interval [CI]: 1.95 - 12.48), non-fatal overdose (AOR = 3.69; 95%CI: 1.45 - 9.39), syringe sharing (AOR = 2.20; 95%CI: 1.12 - 4.32), and female gender (AOR = 0.41; 95%CI: 0.20 - 0.82). Among those who reported a history of incarceration, 59 (29.9%) reported injection drug use in prison, and 48 (81.4%) of these individuals reported sharing syringes in prison. Incarceration was not associated with the number of injections performed in the previous week (*p *= 0.202).

**Conclusion:**

Over three-quarters of the IDU participating in this study reported a history of incarceration, and 30% of these individuals reported injection drug use within prison. Further, an alarmingly high level of syringe sharing within prison was reported, and incarceration was not associated with reductions in drug use. These findings provide further evidence of the need for community diversion strategies, as well as harm reduction programs, in Thai prisons.

## Background

In many countries, drug law enforcement continues to be a dominant societal response to illicit drug use [1,2]. Consequently, injection drug users (IDU) are frequently arrested and incarcerated [3-5]. A large body of evidence indicates that incarceration is associated with elevated risks of drug-related harm among IDU, including the spread of blood-borne pathogens such as human immunodeficiency virus (HIV) and hepatitis C virus (HCV) [6-10]. For example, HIV outbreaks associated with injection drug use in prison have been observed in Scotland [11], Lithuania [12], and the Russian Federation [13]. Endemic sharing of injection equipment has been identified as a major factor contributing to high HCV incidence in prisons in Australia [14] and Scotland [15]. The burden of prison-related epidemics of infectious disease is essentially linked with the public health of communities due to high inmate turnover and the potential spread of prison-acquired infections among non-inmate populations.

The first major wave of HIV infection in Thailand is believed to have occurred among IDU inmates in Bangkok in 1988 [16]. Since then, several studies have suggested that incarceration has continued to be an important risk factor for HIV infection among Thai IDU [17-21]. The HIV prevalence among Thai IDU remains disproportionately high, standing between 30-50% [22-24]. Moreover, a recent study reported an HIV/HCV co-infection rate of 98.8% among HIV-positive IDU prisoners in Bangkok [25]. Despite these findings, Thailand has consistently pursued an aggressive enforcement-based response to illicit drug use [17,26,27]. The inmate population in Thailand has more than tripled between 1992 and 2002, with approximately 70% of these incarceration events attributable to drug-related charges [28,29].

The most aggressive "war on drugs" campaign in Thailand was initiated in 2003 by the former Thai Prime Minister Thaksin Shinawatra, which involved renewing and augmenting efforts to arrest and incarcerate suspected drug users and dealers [26]. This campaign also involved dramatically scaling up compulsory addiction treatment programming [30,31]. In 2008, the Thai government reinstituted this policy initiative [32,33]. While epidemiological evidence has linked incarceration with HIV infection among Thai IDU, little is known about rates of and factors associated with incarceration among IDU in the wake of these intensive law enforcement campaigns. Therefore, we sought to characterize the prevalence and correlates of incarceration among a community-recruited sample of IDU in Bangkok, Thailand with the aim of informing policies specific to addiction and public health.

## Methods

Data for these analyses were obtained from the Mitsampan Community Research Project, a collaborative research effort involving the British Columbia Centre for Excellence in HIV/AIDS (Vancouver, Canada), the Mitsampan Harm Reduction Center (Bangkok, Thailand), the Thai AIDS Treatment Action Group (Bangkok, Thailand), and Chulalongkorn University (Bangkok, Thailand). During July and August of 2008, the research partners designed and undertook a cross-sectional study targeting local IDU in Bangkok recruited through peer-based outreach efforts and word-of-mouth. Invited participants were asked to attend the Mitsampan Harm Reduction Center, where they provided informed consent and completed an interviewer-administered questionnaire. The survey instrument elicited demographic data, information on drug use patterns, HIV risk behavior, overdose experiences, interactions with the criminal justice system (including police and incarceration), and experience with health care. Upon completion of the questionnaire, participants were provided a stipend of 250 Thai baht (about US$7.50) and reimbursed for transportation costs. The study has been approved by the research ethics boards at the University of British Columbia and Chulalongkorn University.

The primary outcome of interest in the present analysis was self-reported history of incarceration (i.e., answering "Yes" to the question: "Have you ever been in prison overnight or longer?"). We compared IDU who reported a history of incarceration with those who did not using univariate statistics and multivariate logistic regression. Variables considered included: median age (< 36.5 years vs. ≥ 36.5 years); gender; education level (≥ secondary school vs. < secondary school); median daily expenses for purchasing drugs (< 300 Thai baht per day vs. ≥ 300 Thai baht per day); ever injected heroin; ever injected *yaba *(methamphetamine); ever injected midazolam; ever injected methadone; ever used drugs in combination; ever shared syringes; ever experienced a non-fatal overdose; ever involved in sex trade; ever in compulsory drug treatment; and ever on methadone treatment. All behavioral variables were coded as yes vs. no.

To examine the bivariate associations between each independent variable and a history of incarceration, we used the Pearson X^2 ^test. Fisher's exact test was used when one or more of the cells contained values less than or equal to five. We then applied an *a priori*-defined statistical protocol that examined factors associated with a history of incarceration by fitting a multivariate logistic regression model that included all variables that were significantly associated with history of incarceration at the *p *< 0.05 level in univariate analyses. Using Pearson's correlation coefficient, we also assessed whether reporting a history of incarceration was associated with reduced drug use (as measured by the number of injections performed in the previous week). All *p*-values were two-sided. In a sub-analysis, we also asked individuals reporting a history of incarceration if they had ever injected drugs and shared syringes in prison.

## Results

In total, 252 IDU were recruited in August 2008, of whom 66 (26.2%) were female. The median age was 36.5 years (IQR: 31.0-46.0 years). The majority of our sample (n = 238; 94.4%) grew up in the Bangkok Metropolitan Area. One hundred fifty-five (61.5%) individuals reported injection heroin use and 86 (34.1%) reported injection *yaba *and *ice *(i.e., methamphetamine) use at least daily in the past six months. Less than half of the participants (n = 111; 44.0%) had ever received methadone treatment, and 116 (46.0%) received drug or alcohol treatment in the past six months. In total, 197 (78.2%) participants reported a history of incarceration.

Table 1 presents the univariate analyses of factors associated with reporting a history of incarceration. As shown, reporting a history of incarceration was positively associated with having been in compulsory drug treatment (odds ratio [OR] = 4.91, 95% confidence interval [CI]: 2.01-12.03), history of non-fatal overdose (OR = 4.40, 95%CI: 1.80-10.79), syringe sharing (OR = 2.44, 95%CI: 1.30-4.58), and midazolam injection (OR = 2.00, 95%CI: 1.08-3.69), and was negatively associated with female gender (OR = 0.48, 95%CI: 0.25-0.91).

**Table 1:** Factors associated with history of incarceration among IDU in Bangkok, Thailand (*n *= 252)

Characteristic	History of incarceration n (%)		OR	95% CI	*p*-value
				
	Yes: 197 (78)	No: 55 (22)			
**Median age**					

< 36.5 years	100 (51)	26 (47)	1.15	0.63 - 2.09	0.647

≥ 36.5 years	97 (49)	29 (53)			

**Gender**					

Female	45 (23)	21 (38)	0.48	0.25 - 0.91	0.024

Other	152 (77)	34 (62)			

**Education**					

≥ Secondary	122 (62)	27 (49)	1.69	0.92 - 3.08	0.089

< Secondary	75 (38)	28 (51)			

**Median daily expenses for purchasing drugs**					

< 300 Thai baht	94 (48)	31 (56)	0.71	0.39 - 1.29	0.258

≥ 300Thai baht	103 (52)	24 (44)			

**Ever injected heroin**					

Yes	186 (94)	48 (87)	2.47	0.91 - 6.70	0.077

No	11 (6)	7 (13)			

**Ever injected *yaba *(methamphetamine)**					

Yes	128 (65)	33 (60)	1.24	0.67 - 2.28	0.497

No	69 (35)	22 (40)			

**Ever injected midazolam**					

Yes	139 (71)	30 (55)	2.00	1.08 -- 3.69	0.027

No	58 (29)	25 (45)			

**Ever injected methadone**					

Yes	33 (17)	6 (11)	1.64	0.65 - 4.15	0.293

No	164 (83)	49 (89)			

**Ever used drugs in combination**					

Yes	141 (72)	34 (62)	1.56	0.83 - 2.91	0.167

No	56 (28)	21 (38)			

**Ever shared syringes**					

Yes	107 (54)	18 (33)	2.44	1.30 - 4.58	0.005

No	90 (46)	37 (67)			

**Ever overdosed**					

Yes	69 (35)	6 (11)	4.40	1.80 - 10.79	0.001

No	128 (65)	49 (89)			

**Ever involved in sex trade**					

Yes	20 (10)	11 (20)	0.45	0.20 - 1.01	0.054

No	177 (90)	44 (80)			

**Ever in compulsory drug treatment**					

Yes	74 (38)	6 (11)	4.91	2.01 - 12.03	< 0.001

No	123 (62)	49 (89)			

**Ever on methadone treatment**					

Yes	89 (45)	22 (40)	1.24	0.67 - 2.27	0.494

No	108 (55)	33 (60)			

Table 2 presents the multivariate analyses of factors independently associated with reporting a history of incarceration. As shown, reporting a history of incarceration was independently and positively associated with having been in compulsory drug treatment (adjusted odds ratio [AOR] = 4.93, 95%CI: 1.95-12.48), ever overdosed (AOR = 3.69, 95%CI: 1.45-9.39), syringe sharing (AOR = 2.20, 95%CI: 1.12-4.32), and was negatively associated with female gender (AOR = 0.41, 95%CI: 0.20-0.82). Among those who reported a history of incarceration, 59 (29.9%) reported injection drug use in prison and 48 (81.4%) of these individuals reported sharing syringes in prison. Reporting a history of incarceration was not associated with the number of injections performed in the previous week (*p *= 0.202).

**Table 2:** Multivariate logistic regression analysis of factors associated with reporting a history of incarceration among IDU in Bangkok, Thailand (*n *= 252)

Characteristic	AOR	95% CI	*p*-value
Gender (female vs. other)	0.41	0.20 - 0.82	0.01

Ever shared syringes (yes vs. no)	2.20	1.12 - 4.32	0.02

Ever overdosed (yes vs. no)	3.69	1.45 - 9.39	0.01

Ever in forced treatment (yes vs. no)	4.93	1.95 - 12.48	< 0.01

## Discussion

More than three-quarters of the community-recruited IDU participating in this study reported a history of incarceration. In multivariate analyses, after adjustment for relevant covariates, reporting a history of incarceration was positively associated with a history of compulsory drug treatment, syringe sharing and non-fatal overdose. Females were less likely than males to have been incarcerated. Among those reporting a history of incarceration, 30% reported injection drug use within prison. Further, an alarmingly high level of syringe sharing (81.4%) was reported among those who injected drugs while in prison. Reporting a history of incarceration was not found to be associated with reduced frequency of injection drug use.

Our findings are consistent with previous studies suggesting that incarceration is very common among Thai IDU [17], and that high levels of drug injection and syringe sharing occur within prisons in this setting [17,34]. However, the proportion of Thai IDU with a history of incarceration observed in this study is considerably higher than the one observed by Beyrer et al. during the period 1999-2000, who reported that 55% of IDU in Northern Thailand had a history of incarceration [17]. This increase in the prevalence of incarceration among Thai IDU may be attributable to differences in sample characteristics or geographic differences in drug law enforcement efforts between Northern Thailand and Bangkok, or the higher prevalence observed in our study may reflect the 2003 war on drugs and subsequent crackdowns that have taken place throughout the country in spite of policy efforts to redirect people who use drugs to treatment centers rather than prison. Regardless, given the high prevalence of HIV infection in this setting [23,24] and the high levels of injection drug use and syringe sharing that occur within prisons, it is unlikely that the epidemic of HIV among Thai IDU can be controlled under the current enforcement-based policy approach. As has been suggested previously [35], controlling or averting epidemics of HIV infection among IDU requires minimizing the transmission of HIV within correctional environments, as well as reducing the rate of incarceration of drug-using populations [36]. Our findings provide further evidence of the urgent need for harm reduction measures, including syringe exchange, in Thai prisons. Indeed, previous evaluations indicate that prison-based syringe exchange programs have been successfully implemented in a range of settings, including low- and middle-income countries [37].

Our findings also suggest that compulsory drug treatment experience is common among Thai IDU with a history of incarceration. This may be due to the fact that Thai IDU are often arrested and held within prison before being diverted to a compulsory treatment setting. Recent reports suggest that IDU spend on average 45 days in prison prior to the diversion [38]. The compulsory treatment system is intended to treat drug users as "patients" and reduce drug-related harms in overcrowded prisons [31,39]. However, given the evidence indicating high rates of syringe sharing among IDU within Thai prisons in our study, as well as in previous studies [19,34], the program of arrest and compulsory drug treatment system, which requires incarceration of IDU, may fail to prevent HIV risk behavior occurring within prisons and thus may serve to perpetuate rather than reduce HIV infection rates among Thai IDU in this setting. Further, the effectiveness of the current system also comes into question given that compulsory drug treatment often consists of a military-style boot camp with no evidenced-based addiction treatment being offered [38]. Taken together with our finding of a lack of an association between incarceration and reduction in injection drug use, our study indicates a need to reform the existing diversion program for drug-using offenders. Unfortunately, the evidence concerning commonly applied diversion programs, such as drug courts, is equivocal [40], and therefore there is a need for innovation in this area. However, as a recent report from the United Kingdom suggested, when diversion programs are applied within a climate shaped by draconian drug policy, such programs tend to be punishment-oriented and inflexible regarding individual treatment needs [41]. Therefore, broader structural changes to the over-arching policy environment are needed to ensure that responses to addiction focus on the health needs of drug users rather than punishment.

We also found that non-fatal overdose was associated with incarceration among Thai IDU. This finding is of significance given the dearth of evidence pertaining to overdose among Thai IDU. While we were unable to establish a temporal relationship between overdose and incarceration in this sample, mounting evidence from Western countries indicates that incarceration greatly exacerbates the risk of fatal and non-fatal overdose among IDU upon release from prison [42-47]. In particular, risk for heroin overdose is likely to be elevated upon release from prisons as a result of reduced tolerance [48]. A recent study from the US reported that drug overdose was a leading cause of death among former inmates [44]. Further research is needed to elicit the linkage between overdose and incarceration among Thai IDU.

This study has several limitations. First, since this is an observational study, we cannot infer causal relationships between exposure and outcome. Second, the study relied on self-report, and therefore the results may be affected by socially desirable reporting. While the peer-administered interviews were expected to minimize such bias, we may still have underestimated the true prevalence of incarceration among Thai IDU or the prevalence of drug use or syringe sharing within prisons. Third, the study sample was not randomly recruited, which means our findings may not be generalizable to other populations of IDU in Thailand. Finally, we could not distinguish incarceration histories by type, duration, or location of incarceration. Future research should aim to address the effect that these modifiers might have on active IDU and their risk behaviors.

## Conclusions

We observed alarmingly high levels of incarceration and syringe sharing in prison among a community-recruited sample of Thai IDU. Incarceration was independently associated with compulsory drug treatment experience, syringe sharing and non-fatal overdose, but was not associated with reductions in the frequency of injecting. These findings raise serious concerns regarding the adverse health consequences resulting from the enforcement-based approach to illicit drug use in Thailand and underscore the urgent need for community diversion programs (e.g., addiction treatment) and harm reduction measures (e.g., syringe exchange) in prisons.

## Competing interests

The authors declare that they have no competing interests.

## Authors' contributions

TK, NF and EW designed the study. CL and TK conducted the statistical analyses. KH and TK drafted the manuscript and incorporated all suggestions. All authors made significant contributions to the conception and design of the analyses, interpretation of the data, and drafting of the manuscript, and all authors approved the final manuscript.

## Pre-publication history

The pre-publication history for this paper can be accessed here:

http://www.biomedcentral.com/1471-2458/9/492/prepub
